# A study of the relationships between *KLF2 *polymorphisms and body weight control in a French population

**DOI:** 10.1186/1471-2350-7-26

**Published:** 2006-03-16

**Authors:** Aline Meirhaeghe, Dominique Cottel, Philippe Amouyel

**Affiliations:** 1 INSERM, U744, Lille; Institut Pasteur de Lille, Lille; Université Lille 2, Lille, France

## Abstract

**Background:**

Factors governing adipose tissue differentiation play a major role in obesity development in humans. The Krüppel-like zinc finger transcription factor KLF2/Lung KLF (LKLF) is a negative regulator of adipocyte differentiation. In this study, we sequenced the human KLF2 gene and several common polymorphisms were found, among them the Pro104Leu and 3'UTR 1239C>A polymorphisms.

**Methods:**

To evaluate the impact of these polymorphisms on anthropometric variables in humans, we genotyped a general population composed of 1155 French individuals (including 232 obese subjects) for these polymorphisms and looked for potential statistical associations with obesity-related variables.

**Results:**

The frequency of the Leu104 and 1239A alleles were 0.22 and 0.18 respectively. Genotype and allele frequencies of the two polymorphisms were comparable in obese, overweight and normal weight subjects. No association between the rare alleles of the polymorphisms and anthropometric variables (BMI, weight, waist and hip circumferences, waist-to-hip ratio and plasma leptin levels) could be detected. Haplotype analyses did not reveal further significant associations.

**Conclusion:**

These data indicate that the Pro104Leu and 3'UTR 1239C>A polymorphisms in *KLF2 *are not associated with obesity and obesity-related traits in humans.

## Background

Obesity is recognized as a worldwide public health problem that may be the major cause of mortality in few years time as it contributes to various severe diseases including type 2 diabetes, atherosclerosis and cancer. The control of adipose tissue differentiation could play an important role in the development of obesity. Different members of several major families, the CCAAT/enhancer-binding protein (C/EBP), peroxisome proliferator-activated receptor γ (PPARγ) and the basic-helix-loop-helix protein ADD1/SREBP1c (adipocyte determination and differentiation factor-1-sterol regulatory element-binding protein-1) are the main orchestrators of this complex pathway [1,2].

The Lung Krüppel Like zinc Finger (LKLF/KLF2) protein is a member of the zinc finger protein family that binds to the consensus sequence 5'-CNCCC-3'. The KLF2 gene was cloned by Anderson and colleagues through the use of the zinc finger domain of EKLF (erythroid Krüppel-like factor) as a bait [3]. The KLF2 gene is located at the 19p13.11-13 locus [4]. KLF2 is expressed in the lung, the developing vascular system, heart, skeletal muscle, kidney, testis, lymphoid tissues and preadipocytes [4,5]. Previous studies demonstrated a critical role for KLF2 in T cell activation and in vascular [6,7]. and lung development [8]. Mice lacking KLF2 die *in utero *between day 11.5 and 13.5 of embryonic life and exhibit retarded growth, craniofacial abnormalities, abdominal bleeding and signs of anaemia [9]. Studies in 3T3-L1 and human primary cells showed that KLF2 is expressed in preadipocytes but not in mature adipocytes. Its overexpresion inhibits adipocyte differentiation, as well as PPARγ, C/EBPα and ADD1/SREBP1c expression [5]. Moreover, Banerjee *et al*. showed that KLF2 can directly inhibit PPARγ2 promoter activity via a KLF2 binding site located at position -82 to -93 bp in the mouse PPARγ2 promoter. This raises the possibility that KLF2 might be involved in body weight control in humans. In order to evaluate the importance of KLF2 in body weight control in humans, we searched for genetic polymorphisms in the gene and looked for their associations with anthropometric variables in a representative sample of the northern French population (n = 1155).

## Methods

### Population study

Within the framework of the WHO-MONICA (Multinational MONItoring of trends and determinants of CArdiovascular diseases) project [10,11], we constituted in 1995–1997 an age (35–64 years)- and gender-stratified sample of men (n = 601) and women (n = 594) living in the Urban Community of Lille in northern France. This study was randomly sampled from the electoral rolls. To our knowledge, there were no related subjects in the sample. The Ethics Committee of the Centre Hospitalier et Universitaire de Lille approved this study and informed consent was obtained from each individual. A detailed questionnaire was completed, which included an evaluation of alcohol and smoking consumption and a personal medical history. Body mass index (BMI), waist-to-hip ratio (WHR) and blood pressure were measured. Genomic DNA was available for 1155 subjects. From this sample, 232 subjects were obese (BMI ≥ 30 kg/m^2^). The level of physical activity was defined as: walking or cycling 15 min or more per day, and/or lifting or carrying heavy objects at work daily, and/or doing sport or physical exercise more than 2 hours a week. Current cigarette smokers were defined as subjects reporting at least one cigarette per day. Total alcohol intake was expressed as the sum of ml of alcohol per week from wine, beer, cider and spirits.

### Sequencing of the KLF2 gene

The KLF2 gene was amplified and sequenced using the following oligonucleotides: For the exons 1 and 2: 5'-ATGGCGCTGAGTGAACCC-3' (position 1 of the ATG) and 5'-CATCTGCGCACGCACACAG-3' (end of exon 2). The corresponding PCR (1109 bp) was then sequenced with these oligonucleotides as well as the intermediate oligonucleotides 5'-GCGGCCTGCAGGAGGTGAG-3' (end of exon 1), 5'-CTGCAGACTCAGGAGAGG-3' (position 98 in intron 1) and 5'-GCGGCTTCGTGCATGCGA-3' (position 390 in exon 2). For the exon 3: 5'-GGTGAGGATCCGGATTGT-3' (position 639 in intron 2) and 5'-GGATCGAGGCTTGTGATGC-3' (position 289 after the stop codon).

### Genotyping

#### Pro104Leu polymorphism

The region encompassing the polymorphism was amplified using the following oligonucleotides: sense 5'-GCGCCGAACCCGAGTCCG-3' and antisense 5'-GCGGTCCACGGGTCAGCC-3'. The PCR reaction was performed using 1 mM MgCl_2 _at an annealing temperature of 60°C. The PCR product (348 bp) was digested with 1 unit of *BseY*I (New England Biolabs, Hertfordshire, UK). The digestion product was resolved on 2% agarose gels. The Leu104 allele is cut by *BseY*I into 2 fragments of 224 and 124 bp whereas the Pro104 allele is not cut.

#### 3'UTR 1239C>A polymorphism

A forced enzymatic restriction site for the *Hae*III enzyme was introduced into the forward primer 5'-ACGACGCCACCACCCCGGC-3' and was used with the reverse primer 5'-GCATCACAAGCCTCGATCC-3' to generate a 157 bp amplicon. The annealing temperature of the primers was 66°C and 2 mM MgCl_2 _was used in the PCR reaction. The PCR product was then digested by 1 unit of *Hae*III (New England Biolabs, Hertfordshire, UK) and the digestion product was separated on a 14% acrylamide gel (acrylamide, bis-acrylamide 19:1). *Hae*III cuts the PCR into 3 fragments in case of the 1239C allele (17+20+120 bp) whereas 2 fragments are generated (37+120 bp) in case of the 1239A allele.

### Statistical analyses

#### Genotype associations

Allele frequencies were estimated by gene-counting and departure from Hardy-Weinberg equilibrium within the study groups was tested using a χ^2 ^test. Three groups of BMI were considered according to WHO definitions: normal weight (BMI<25 kg/m^2^), overweight (25 ≤ BMI<30 kg/m^2^) and obese (BMI ≥ 30 kg/m^2^) *KLF2 *genotype effects were tested by gender on anthropometrical variables by analysis of variance (procedure GLM) with and without adjustment for covariates (age, physical activity, smoking and alcohol consumptions) using a dominant model (heterozygotes combined with homozygotes for the rare allele versus homozygotes for the common allele). Leptin values were log-transformed to obtain normal distributions. Throughout, *p*-values < 0.05 were interpreted as significant. All genotype analyses were carried out with the SAS software (version 8, SAS Institute, Cary, NC). Power calculations were based on a two-sided, two-sample (dominant model), t-test, with a power of 0.80, an α of 0.05, and the means and standard deviations of BMI displayed in tables 3 and 4, in men and women respectively.

#### Haplotype analyses

were based on the maximum likelihood model described in [12] and linked to the SEM algorithm [13] implemented in the THESIAS program [14]. By definition, the reference haplotype corresponds to the more frequent haplotype.

## Results

The human KLF2 gene is composed of three exons and two introns. Exons 1, 2 and 3 as well as all intron 1 and the last 183 bp of intron 2 were sequenced in 10 (5 obese and 5 non-obese) subjects. Three polymorphisms were detected: a C/T substitution in intron 1 (rs7248864, 75+44C>T, T allele frequency <10%), a C/T polymorphism in exon 2 replacing a proline with a leucine at codon 104 (rs3745318, Pro104Leu, Leu104 frequency around 20%), and a C/A polymorphism in the 3'UTR 1239 nucleotides after the first coding nucleotide (or 171 nucleotides after the STOP codon) (rs15336, 1239C>A, 1239A allele frequency around 15%). These polymorphisms, by the end of this study, were all displayed on the NCBI dbSNP web site. We were unable to amplify and sequence the proximal promoter of *KLF2 *(up to 400 bp upstream the ATG) despite attempts with several pairs of primers. However, one SNP (rs8106384, -18T>C) in the 5'UTR (18 bp before the ATG) was displayed on the NCBI dbSNP web site. We tested for the existence of this polymorphism but found only one heterozygote in 59 individuals (-18T allele frequency < 1%). We therefore decided to genotype the Lille population study for the Pro104Leu and 3'UTR 1239C>A polymorphisms as they were frequent enough to give sufficient statistical power and were more likely to have a functional impact at the protein or gene expression level.

There were 60.8% of *Pro104Pro*, 34.0% of *Pro104Leu *and 5.2% of *Leu104Leu *individuals (Leu104 allele frequency = 0.22) and 67.6% of 1239CC, 29.6% of 1239CA and 2.8% of 1239AA individuals (1239A allele frequency = 0.18) in the population. These distributions were not different from the frequencies expected in a population in Hardy-Weinberg equilibrium. The two polymorphisms were in partial linkage disequilibrium, the Pro104 allele being associated with the 1239A allele (D' = -0.75, p < 0.0001) whilst the two SNPs did not give the same information (r^2 ^= 0.035) (table 1).

**Table 1:** Linkage disequilibrium between the Pro104Leu and 3'UTR 1239C>A polymorphisms

		Pro104Leu			
		**Pro104Pro**	**Pro104Leu**	**Leu104Leu**	**Total**
			
**3'UTR 1239C>A**	**CC**	421 (37.4)	284 (25.2)	54 (4.8)	759
	**CA**	233 (20.7)	97 (8.6)	4 (0.4)	334
	**AA**	28 (2.5)	4 (0.4)	0 (0)	32
**Total**		682	385	58	1125

Data are n (%).

D' = -0.75 and r^2 ^= 0.035.

Only subjects with genotypes available for both polymorphisms are represented in the table.

Because anthropometric variables and obesity status differ significantly according to gender, we assessed the impact of the *KLF2 *polymorphisms in men and women separately in the analyses. The genotype and allele distributions for both polymorphisms were similar in lean (BMI<25 kg/m^2^), overweight (25 ≤ BMI>30 kg/m^2^) or obese (BMI ≥ 30 kg/m^2^) individuals in men and women independently (table 2).

**Table 2:** Genotype and allele frequencies of the Pro104Leu and 3'UTR 1239C>A polymorphisms according to obesity status by gender.

	**Men**				**Women**			
		
	**Normal weight**	**Overweight**	**Obese**	p*	**Normal weight**	**Overweight**	**Obese**	p*
		
Pro104Pro, n (%)	140 (60.9)	147 (60.8)	61 (59.2)		153 (59.5)	103 (59.2)	83 (65.4)	
Pro104Leu, n (%)	83 (36.1)	78 (32.2)	37 (35.9)	0.36	90 (35.0)	60 (34.5)	40 (31.5)	0.67
Leu104Leu, n (%)	7 (3.0)	17 (7.0)	5 (4.9)		14 (5.5)	11 (6.3)	4 (3.1)	
Total	230	242	103		257	174	127	
		
1239CC, n (%)	162 (69.5)	158 (66.1)	67 (65.1)		176 (68.8)	119 (68.4)	84 (65.6)	
1239CA, n (%)	62 (26.6)	72 (30.1)	34 (33.0)	0.69	74 (28.9)	53 (30.5)	40 (31.3)	0.78
1239AA, n (%)	9 (3.9)	9 (3.8)	2 (1.9)		6 (2.3)	2 (1.1)	4 (3.1)	
Total	233	239	103		256	174	128	

* p values for the 3*3 analysis.

Normal weight: BMI < 25 kg/m^2^, overweight: 25 ≤ BMI < 30 kg/m^2^, obese: BMI ≥ 30 kg/m^2^.

**Table 3:** Impact of the Pro104Leu polymorphism on obesity-related traits by gender.

	**Men**				**Women**			
		
n	**Pro104Pro 348**	**Pro104Leu 198**	**Leu104Leu 29**	p*	**Pro104Pro 341**	**Pro104Leu 190**	**Leu104Leu 29**	p*
		
Weight, kg	80.0 ± 13.2	79.0 ± 14.1	83.9 ± 10.9	0.77	69.1 ± 15.0	68.8 ± 15.9	67.7 ± 15.2	0.76
BMI, kg/m^2^	26.6 ± 4.1	26.5 ± 4.3	27.5 ± 2.5	0.91	26.8 ± 5.7	26.7 ± 6.0	25.5 ± 4.9	0.70
Waist, cm	96.1 ± 10.9	95.8 ± 11.1	97.8 ± 7.9	0.98	86.1 ± 14.6	86.0 ± 14.9	84.7 ± 13.7	0.83
Hip, cm	101.7 ± 7.5	101.2 ± 7.4	102.0 ± 5.6	0.59	104.0 ± 12.1	104.2 ± 12.5	103.1 ± 14.5	0.93
WHR	0.94 ± 0.07	0.95 ± 0.07	0.96 ± 0.07	0.54	0.83 ± 0.08	0.82 ± 0.08	0.82 ± 0.08	0.59
Leptin, ng/ml	9.5 ± 7.8	8.8 ± 7.1	9.8 ± 6.7	0.78	24.1 ± 14.1	23.3 ± 13.9	24.6 ± 14.4	0.64

* for the comparison between Pro104Pro vs Leu/* subjects.

**Table 4:** Impact of the 1239C>A polymorphism on obesity-related traits by gender

	**Men**				**Women**			
		
n	**CC 387**	**CA 168**	**AA 20**	p*	**CC 381**	**CA 167**	**AA 12**	p*
		
Weight, kg	79.7 ± 13.4	80.1 ± 13.8	78.9 ± 12.1	0.85	69.2 ± 16.0	68.6 ± 13.6	68.3 ± 12.4	0.67
BMI, kg/m^2^	26.5 ± 4.1	26.8 ± 4.2	25.5 ± 3.6	0.62	26.7 ± 6.0	26.6 ± 5.4	26.9 ± 5.2	0.85
Waist, cm	96.0 ± 10.8	96.5 ± 10.9	93.1 ± 9.4	0.85	86.1 ± 15.2	85.9 ± 13.3	90.6 ± 13.0	0.97
Hip, cm	101.4 ± 7.1	102.0 ± 8.0	100.8 ± 6.2	0.48	104.1 ± 12.9	104.0 ± 11.3	104.0 ± 10.9	0.92
WHR	0.95 ± 0.07	0.95 ± 0.07	0.92 ± 0.07	0.72	0.83 ± 0.08	0.82 ± 0.08	0.87 ± 0.08	0.75
Leptin, ng/ml	9.4 ± 7.3	9.0 ± 8.2	7.5 ± 4.9	0.13	24.0 ± 14.3	23.5 ± 13.5	28.6 ± 14.7	0.93

* for the comparison between CC and CA+AA subjects

We then looked for potential associations between the rare (Leu104 and 1239A) alleles of the polymorphisms and anthropometric variables (body weight, BMI, waist and hip circumferences, WHR, plasma leptin levels). No significant differences could be detected between Pro104Pro individuals and Leu104 allele bearers or between 1239CC and 1239A allele bearers concerning these phenotypes (tables 3 and 4) even after adjustment for age, smoking and alcohol consumption and physical activity level, in either men or women.

We also performed haplotype analyses in order to study allele combinations. The frequency of the haplotype comprising the 2 common alleles (Pro104-1239C) was around 60%. The frequencies of the 4 possible haplotypes were similar in obese and non-obese subjects (table 5). No significant global effect of haplotypes could be detected in either men or women for any quantitative variables considered (body weight, BMI, waist and hip circumferences, WHR or plasma leptin levels) (data not shown).

**Table 5:** Frequencies of haplotypes according to obesity status in men and women.

	**Men**			**Women**		
		
	**Non-obese (466)**	**Obese (103)**	***p***	**Non-obese (423)**	**Obese (127)**	***p***
		
Pro104-1239C	0.614	0.599		0.602	0.624	
Leu104-1239C	0.205	0.217	0.97	0.231	0.187	0.48
Pro104-1239A	0.165	0.173		0.164	0.187	
Leu104-1239A	0.016	0.011		0.003	0.002	

p: p value for global haplotype effect.

## Discussion

To our knowledge, this is the first time the study of polymorphisms in the KLF2 gene have been undertaken. Following the observations from Banerjee *et al*. who showed KLF2 is a negative regulator of adipocyte differentiation and that it directly inhibits PPARγ [5], one of the key regulators of adipose tissue differentiation, we hypothesised that KLF2 may be a candidate gene for body weight and fat mass control in humans. We identified several polymorphisms in *KLF2 *and genotyped the Lille population study for two of them, one in the coding region (Pro104Leu) and the other in the 3' untranslated region (1239C>A) of the gene. We could not observe any significant association between the polymorphisms and obesity status or anthropometric variables in our sample, by individual or haplotype analyses.

The KLF2 protein is rich in proline residues (17.5% of the total residues) and form Pro-rich repeats of three to eight amino acids in a row [4]. Conkright *et al*. showed amino acids 1–100 of KLF2 correspond to the activation domain of the protein [15]. The polymorphism at codon 104 (Pro>Leu) is located outside the activation domain but it replaces a proline by a leucine and might therefore modify the structure and the activity of the protein. However, when comparing rodent and human sequences, a glutamine residue is present at codon 104 in the murine and rat sequences instead of a proline residue in humans, suggesting this amino acid is probably not crucial for the protein function. Because the 1239C>A polymorphism is located into the 3' untranslated region of *KLF2*, it could potentially modify the gene expression or mRNA stability but this remains to be determined. Also, it remains possible these polymorphisms are in linkage disequilibrium with other functional variant(s) nearby that remains to be detected.

Our study has both strengths and weaknesses. It was conducted in a large random sample of population which avoids possible bias due to recruitment in hospitals or clinics and university staff or students. The analyses were performed in men and women separately which is an important advantage with regard to the impact of gender on fat mass depots and obesity risk. A difference in BMI between genotype groups should be at least of 1 kg/m^2 ^to be considered phenotypically relevant. The population-study we used was powerful enough (power ≥ 80%, α = 5%) to detect a difference in BMI of 1.0 and 1.4 kg/m^2 ^in men and women respectively for both polymorphisms. The major limitation of the study may be that only two SNPs in *KLF2 *were studied and therefore we can not totally exclude that other SNPs in or nearby (especially in regulatory regions) the KLF2 gene might be associated with obesity traits.

## Conclusion

In conclusion, the Pro104Leu and 1239C>A polymorphisms in *KLF2 *were not associated with obesity and obesity-related traits in a large representative sample of French men and women.

## Competing interests

The author(s) declare that they have no competing interests.

## Authors' contributions

AM genotyped the population samples, analysed the data and wrote the paper.

DC and PA enrolled the participants and contributed to writing the paper.

## Pre-publication history

The pre-publication history for this paper can be accessed here:


